# Between the retirement of phenytoin and the crowning of levetiracetam: balancing innovation, safety, accessibility and well-established practices

**DOI:** 10.1055/s-0046-1817050

**Published:** 2026-03-11

**Authors:** Lécio Figueira Pinto

**Affiliations:** 1 Universidade de São Paulo, São Paulo SP, Brazil.

Convulsive status epilepticus (CSE) is one of the most critical neurological emergencies. Benzodiazepines remain the first-line therapy; however, their underuse and suboptimal dosing in the real-world practice remain persistent challenges. 1 2 Once benzodiazepines fail, clinicians must promptly choose a second-line antiseizure medication—a decision historically dominated by phenytoin (PHT) but increasingly challenged by levetiracetam (LEV).

## PHENYTOIN: THE LEGACY DRUG

For decades, PHT has been the cornerstone of second-line therapy after benzodiazepine failure. Its efficacy in terminating seizures is well established, and its widespread availability and low cost have made it indispensable in many regions. 3 However, the Established Status Epilepticus Treatment Trial 4 (ESETT) demonstrated that PHT, LEV, and valproic acid are similarly effective in benzodiazepine-resistant CSE, with no significant differences in seizure cessation or early adverse events.

Nonetheless, this trial's 4 pragmatic design leaves certain limitations. The primary endpoint, the absence of clinically-evident seizures and improved consciousness at 60 minutes, does not account for delayed adverse events, drug interactions, or the practical challenges of drug administration and monitoring. Moreover, PHT's narrow therapeutic window, complex pharmacokinetics, and potential for idiosyncratic toxicity have long been recognized. 5 6 Severe cutaneous reactions such as Stevens-Johnson syndrome and toxic epidermal necrolysis occur more frequently with PHT than with most other antiseizure drugs. 7

Intravenous PHT administration also requires careful dose calculation, an in-line filter, slow infusion under cardiac monitoring, and vigilance for arrhythmias and hypotension—conditions that are difficult to maintain in emergency settings. 5 While its familiarity among physicians and affordability justifies its continued use in many hospitals, these safety concerns and operational difficulties increasingly limit its suitability for rapid, generalized emergency use.

## LEVETIRACETAM: THE MODERN CONTENDER

Levetiracetam represents a paradigm shift toward safer, simpler, and more predictable antiseizure therapy. Multiple randomized controlled trials, including the ESETT, 4 the Levetiracetam versus phenytoin for second-line treatment of convulsive status epilepticus in children 8 (ConSEPT), and the Emergency Treatment with Levetiracetam or Phenytoin in Status Epilepticus in Children 9 (EcLiPSE), have shown LEV to be as effective as PHT for benzodiazepine-resistant CSE. Pediatric trials particularly highlight LEV's favorable tolerability and fewer serious adverse events compared with PHT. 8 9

A meta-analysis 10 including 11 randomized clinical trials confirmed that LEV offers at least comparable efficacy and fewer major adverse events than PHT in established status epilepticus (SE). In children, another systematic review 11 reported faster seizure cessation, shorter Intensive Care Unit (ICU) stays, and fewer complications with LEV. Beyond clinical efficacy, LEV's pharmacological profile, with linear kinetics, minimal drug interactions, and absence of need for serum-level monitoring makes it ideal for acute management and seamless transition to oral therapy. Rapid intravenous administration, even of undiluted doses of up to 4,500 mg, has been proven to be safe and well tolerated. 12

Although LEV may cause behavioral adverse effects, such as irritability and mood disorders, these are not relevant during the acute emergency treatment. In contrast, PHT's adverse events, such as those of cardiac and cutaneous natures, can be life-threatening and require immediate intervention. Moreover, cost-effectiveness analyses 13 14 have shown that, when the expenses of monitoring and managing PHT-related complications are considered, LEV becomes economically competitive.

## BRIDGING INNOVATION AND ACCESSIBILITY

Transitioning from PHT to LEV as the standard second-line agent for SE is evidence-based and logical, but not without barriers. The higher acquisition cost and limited availability of LEV in public hospitals in low- and middle-income countries remain major obstacles. 14 Moreover, PHT's deep-rooted familiarity among emergency physicians continues to influence prescription patterns.

A rational transition should not imply abrupt abandonment of PHT, but rather an organized shift supported by institutional policy, education, and accessibility measures. Hospitals should prioritize incorporating LEV into emergency forms, and training programs must equip physicians to use it confidently and correctly. Negotiations with suppliers and public health authorities are crucial to reduce costs and ensure broader access.

Phenytoin deserves recognition for its historical contribution to the management of SE, especially where resources are limited. However, LEV's safety profile, ease of administration, and efficacy that is at least equivalent make it the preferred agent for most modern emergency settings. The ongoing “retirement” of PHT is not a dismissal of its value, but an evolution toward safer and more practical seizure management, in which innovation and accessibility must coexist.
